# Complications after direct anterior versus Watson-Jones approach in total hip arthroplasty: results from a matched pair analysis on 1408 patients

**DOI:** 10.1186/s12891-019-2463-x

**Published:** 2019-02-14

**Authors:** Antonio Klasan, Thomas Neri, Ludwig Oberkircher, Dominik Malcherczyk, Thomas Jan Heyse, Christopher Bliemel

**Affiliations:** 1 University Hospital Marburg, Center for Orthopedics and Traumatology, Baldingerstrasse, 35043 Marburg, Germany; 2 Schwarzwald Baar Clinic, Department for Orthopedics, Sonnhaldenstr. 11, 78166 Donaueschingen, Germany; 3 Department for Orthopedics, University Hospital St. Etienne, Avenue Albert Raimond, 42270 Saint-Priest-en-Jarez, France; 4 Orthomedic Frankfurt Offenbach, Herrnstraße 57, 63065 Offenbach, Germany

**Keywords:** Hip arthroplasty, Direct anterior approach, Anterolateral approach, Bleeding, Infection

## Abstract

**Background:**

The direct anterior approach (DAA) has gained popularity in total hip arthroplasty (THA) over the past decade. A large number of studies have compared the DAA to other approaches with inclusion of a learning curve phase. The aim of this study was to compare the complication rate and bleeding between the DAA and the anterolateral approach after the learning curve phase.

**Methods:**

For this retrospective, single-institutional study, propensity score matching was performed, from an initial cohort of 1408 patients receiving an elective THA. Two matching groups were created, comprising of 396 patients each. After matching, both groups were similar in age, gender, body mass index, anesthesiologist’s score and surgeon’s experience.

**Results:**

Average age in the matched groups was 68.7 ± 10.3 years. The total blood loss was similar in both groups, 450 vs 469 mL (*p* = 0.400), whereas the transfusion rate (14.1 vs 5.8%, *p* < 0.001) and the overall complication rate (17.6 vs 12.1%, *p* = 0.018) were lower in the DAA group. The overall fracture rate was comparable, 1.5 vs 1% (*p* = 0.376), as well as the early infection rate, 0.3 vs 1% (*p* = 0.162). The dislocation rate was significantly increased in the DAA group, 2.2 vs 0.5% (*p* = 0.032).

**Conclusions:**

The direct anterior approach has comparable short-term surgical complications with reduced transfusion and general complication rates.

**Level of evidence:**

Level III retrospective study.

## Background

The direct anterior approach (DAA) was first described in 1881, when Marburg born surgeon Carl Hueter reported it to facilitate the femoral head resection in septic coxitis [1]. It gained increasing popularity in the nineteen fifties through the work of the Norwegian born American surgeon Smith-Petersen [2]. Further modifications of this approach have been developed in order to facilitate the detection of the Hueter interval [3]. The newest iterations using an improved orthopedic table [4] report less blood loss and pain [5, 6]. Also, shorter in-hospital stays [7, 8] with similar dislocation rates [7] have been reported.

A systematic review on total hip arthroplasty conducted in 2009 has shown that minimally invasive surgery in general reduces blood loss and has similar outcome [9] whereas other studies have shown that the complication rate is higher due to poorer exposure [10–13]. Another recent prospective study on a minimally invasive anterior approach, including the learning curve phase, shows a reduced blood loss but a slightly increased complication rate [14]. Infection rates have been shown to be similar [15].

It has also been shown that potential benefits of minimal invasive surgery in total hip arthroplasty (THA) are usually limited to several weeks following surgery [16]. A problem of many of the earlier studies reporting on potential benefits of the DAA is the inclusion of an initial learning curve. This leaves the question if there is a difference after a given learning curve phase unanswered.

Therefore, the purpose of the present study was to directly compare a DAA and an anterolateral approach with regards to bleeding and perioperative complications as primary endpoints. It was hypothesized that THA performed via DAA would reduce perioperative complications and blood loss.

## Methods

### Cohort demographics and matching

**Fig. 1:**
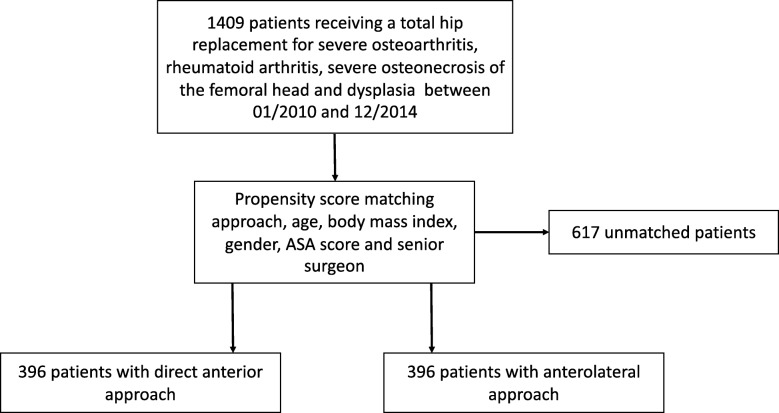
Patient selection flow chart

For this single-institutional study, a total of 1408 patients that underwent THA were included for a retrospective data collection. The recruitment period was between January 2010 and December 2014. The institution at which the study was conducted is a high-volume orthopedic teaching hospital, where approximately 350 primary and 50 revision THA are done yearly. The inclusion chart is shown on Fig. 1. Inclusion criterion was an elective THA due to osteoarthritis after failed conservative treatment, severe osteonecrosis of the femoral head, rheumatoid arthritis and dysplasia. Fractures were excluded. Propensity score matching for the approach analysis was performed using the caliper technique with the caliper set at 0.2, using the following criteria: patient age at surgery, body mass index (BMI), gender, ASA scores, and surgery performed by one of the three senior surgeons. Senior surgeons (TL, LP and RH) were the three most experienced surgeons in the clinic, each performing at least 100 primary arthroplasties a year at the time of the study, for at least 3 years in each surgeon’s case. The standard approach of the institution at the time of the study was the DAA. The criteria for choosing the approach were instrument availability and teaching purposes. The intra-surgeon distribution of approaches was without significant difference to the overall distribution of the clinic (*p* = 0.85) and there was no difference in number of procedures with each approach over the years.

### Surgical information

A standard, preoperative digital planning was performed using the mediCAD© (MediCAD, Atlanta, Georgia, U.S.) software based on the preoperative anterior-posterior pelvis x-ray, in order to facilitate implant and size choice. Both DAA and anterolateral Watson-Jones approach were equally used at the study center by the three senior surgeons for over seven years prior to patient enrolment. With the DAA, a cementless Versafit Trio Cup (Medacta, Castel San Pietro, Ticino, Switzerland) and either a cementless Quadra© or AMI© Stem (both Medacta, Castel San Pietro, Ticino, Switzerland) were used. The implants were used in combination with the Medacta extension table, developed specifically to be used with these implants and the DAA approach. For the anterolateral approach, the Watson-Jones approach was used. All of these patients received an R3 Cup in combination with a cementless SBG stem (Smith & Nephew, London and Hull, United Kingdom). All patients received a 28 mm ceramic head (Biolox Delta©, Ceramtec, Plochingen, Germany). Fluoroscopy was used in all of the procedures. Tranexamic acid was not used during the period for which the data has been collected.

Patients receiving the anterolateral approach had one drainage placed subfascially and one epifascially. Patients treated via DAA received one drainage subfascially. Drainages remained in place until the second postoperative day, at which the first wound control was done. All intraoperative and postoperative complications as well as transfusions were documented digitally in the patient history chart.

### Outcome parameters

All complications were digitally recorded in the patients’ charts and reviewed for this study. Data from the in-hospital stay was analyzed for the purpose of this study. Low molecular weight heparin (enoxaparin, Clexane©, Sanofi) was prescribed for deep venous thrombosis prophylaxis for six weeks postoperatively. According to the hospital’s standard procedure, transfusion criteria were either a postoperative haemoglobin (Hb) level lower than 7 g/dl or 8 g/dl, with the patient being haemodynamically symptomatic. Haemoglobin and haematocrit were measured 24 h, 48 h and five days postoperatively. All infusions and transfusions were measured and documented. Blood loss was calculated as described by Charrois et al. [17]: Total blood loss (ml of erythrocytes: 100% haematocrit) = compensated blood loss + non-compensated blood loss; compensated blood loss (ml) = number red blood cell units x ml red blood cells (RBC) per red blood cell unit (300 ml per unit); non-compensated blood loss (ml): = total blood volume x (preoperative haematocrit - postoperative haematocrit); total blood volume (ml): in men = 604 + 0.0003668 x [height (cm)]^3^ + 32.2 x weight (kg); in women = 183 + 0.000356 x [height (cm)]^3^ + 33 x weight (kg).

Perioperative complications were defined according to the Dindo classification [18]. Nevertheless, due to a lack of relevance we concentrated on orthopedically relevant surgical complications which were recorded separately as intra- and postoperative fractures and dislocations. Surgical site infection was defined according to the CDC criteria [19]. As the largest joint replacement center in the region, all patients having a major surgical complication after discharge were readmitted to our clinic.

Statistical analyses were performed using SPSS (Version 23.0, SPSS, Chicago, IL, USA) and Microsoft Office Excel 2017 (Microsoft Corporation, Seattle, USA). Continuous variables were presented with mean and range, whereas group variables are presented with numbers and percentages.

Continuous variables were analysed using t tests, and multiple one-way analysis of variance (ANOVA) were used for nominal variables, supplemented by Bonferroni post-hoc and Chi-squared tests. Statistical significance was set at *p* < 0.05.

## Results

### Propensity score matching results

The patient demographics before and after propensity score matching are shown in Table 1. Mean patient age in the initial cohort was 68.9 ± 10.4 years. There were 811 women and 598 men in the baseline population. Sixty percent of all THA were performed using DAA and 40% using Watson Jones. The main indication for surgery was osteoarthritis in 1370 patients, rheumatoid arthritis in 25 patients, severe osteonecrosis of the femoral head in 8 patients and hip dysplasia in 5 patients. Average BMI was 27.8 ± 4.8. Before matching, all parameters except gender were statistically different. However, after matching the patients, two groups, each consisting of 396 patients were created, without a significant difference in all of the considered parameters.

**Table 1:** The demographic data

	Pre-matched cohort				Post-matched cohort			
	Total	DAA^b^	Watson-Jones	*p* value	Total	DAA^b^	Watson-Jones	*p* value
Number of patients	1408	846 (60.0%)	562 (39.9%)		792	396	396	
Age (years)	68.9 (± 10.4)	67.4 (± 10.3)	71.4 (± 10)	< 0.001	68.7 (± 10.3)	67.3 (±10.3)	68.5 (±10.2)	0.198
BMI^a^	27.8 ± (4.8)	27.2 (± 4.9)	28.7 (± 5.5)	< 0.001	28.1 (± 4.9)	27.7 (±4.7)	27.6 (±4.9)	0.245
Female	811 (57.5%)	476 (56.3%)	335 (59.5%)	0.214	486 (61%)	243 (61.3%)	243 (61.3%)	0.382
ASA** I	98 (6.9%)	78 (9.2%)	20 (3.6%)		40 (5.0%)	24 (6.1%)	16 (4.0%)	
ASA** II	934 (66.3%)	603 (71.2%)	331 (58.9%)		568 (71.2%)	284 (71.7%)	284 (71.7%)	
ASA** III	374 (26.5%)	164 (19.3%)	210 (37.3%)	< 0.001	184 (23.2%)	88 (22.22%)	96 (24.2%)	0.175
ASA** IV	2 (0.1%)	1 (0.1%)	1 (0.1%)		0 (0.0%)	0 (0.0%)	0 (0.0%)	

^a^*BMI* body mass index

^b^*DAA* direct anterior approach

**ASA American Society of Anesthesiologists’ score

### Comparison between the groups

The results are shown in Table 2. Perioperative, non-compensated blood loss was slightly lower in the antero-lateral approach, although not significantly different (*p* = 0.126). The transfusion rate was significantly higher with the anterolateral approach (14.1% vs. 5.8%, *p* < 0.001) at an overall transfusion rate of 9.9%. Although the transfusion rate was higher, the total blood loss was without significant difference (*p* = 0.400).

**Table 2:** Results of the comparison between the groups

	DAA*	Watson-Jones	*p* value
Number of patients	396	396	
Non-compensated blood loss (mL)	387 (± 163)	405 (± 170)	0.126
Transfusion rate	23 (5.8%)	56 (14.1%)	**< 0.001**
Total blood loss (mL)	450 (± 362)	469 (± 292)	0.400
Complication rate	48 (12.1%)	70 (17.6%)	**0.018**
Intraoperative fracture rate	2 (0.5%)	2 (0.5%)	0.688
Postoperative fracture rate	4 (1%)	2 (0.5%)	0.343
Overall fracture rate	6 (1.5%)	4 (1%)	0.376
Dislocation rate	9 (2.2%)	2 (0.5%)	**0.032**
Surgical site infection rate	1 (0.3%)	4 (1%)	0.162

*DAA direct anterior approach

The general complication rate was higher in the Watson-Jones group (*p* = 0.018). There was no significant difference in the incidence of life-threatening complications occurring in this study (level IV according to the Dindo classification): pulmonary embolism (*p* = 0.318), myocardial infarction (*p* = 0.564), cardiovascular complications (*p* = 0.245) and ileus (*p* = 0.705). In terms of fractures (*p* = 0.376) and early infection rate (*p* = 0.162), no significant differences were observed.

In the DAA group, two intraoperative fractures occurred. One of these was a femoral fissure Vancouver B that was intraoperatively treated with wiring and the other fracture was a Vancouver A type fracture, treated conservatively. Also, in the group of patients treated by the Watson-Jones approach, two intraoperative fractures occurred. One was a minimally dislocated acetabular fracture that needed no revision and the other was a Vancouver B fracture that was treated with immediate wiring. Fractures during hospital stay occurred a total of six times, four times in the DAA group and two in Watson-Jones group without significant difference between the two groups (*p* = 0.343). All patients required revision, either with wiring or revision of the stem or cup.

Dislocation rate was significantly higher in the DAA group (*p* = 0.032) with a total of nine patients. Four of these patients received a longer offset head; the others did not require surgery. In the Watson-Jones group, two patients experienced a dislocation and both were treated surgically with a higher offset head.

Finally, the surgical site infection rate was higher in the Watson-Jones group. In all cases it was a superficial infection. Only one patient from the Watson-Jones group needed epifascial revision surgery; all other patients were treated with antibiotics. In the DAA group, all patients could be treated conservatively. None of the patients needed implant revision due to early infection.

## Discussion

The current propensity score matched study aimed to compare the DAA and the anterolateral approach in terms of complication rate and bleeding. The principal findings revealed that DAA showed a decreased transfusion and general complication rate, comparable orthopedic complications according to the Dindo classification and an increased dislocation rate when compared to the anterolateral approach.

Mean patient age and men:women ratio of this cohort correspond to those reported for THA in the literature [20], confirming a comparable group. The total blood loss observed is similar to reported values in the literature as well [16].

Overall, the transfusion levels observed in this study are still low compared to large sample studies reporting transfusion rates of up to 20% [21]. Within our cohort, the transfusion rate for the DAA was significantly lower. This could be due to the higher number of drains routinely used, but also due to the overall bigger exposure of the hip joint with the anterolateral approach. These differences have been observed in a recent literature review [22]. This is an important finding since transfusions have known risks for the patients [23]. There are protocols in place that reduce the transfusion incidence, although their widespread use is still lacking [24].

As described by Dindo et al., defining complications after surgery can be difficult since the minor, non-surgical complications are not of primary importance for the orthopedic surgeon [18]. In this study, even though a difference in overall complications has been observed, the incidence of all life threating complications was without significant difference. The difference is due to a higher complication rate of minimal complications, such as a simple urinary tract infection [25] or a slight hypokalemia [26]. A far more important finding is the similar rate of surgical complications, especially considering other studies mostly favor standard approaches [16, 20], regardless of the presence of the learning curve [27]. The lack of differences between the two groups suggests that experienced surgeons have a low fracture rate altogether, regardless of the approach [20, 28, 29]. The surgical site infection rate was within the lower margin of a meta analyses conducted by de Geest et al. [20], and a lack of a difference has been previously observed [15].

The only difference in surgical complications was the higher dislocation rate for DAA, which was higher than in comparable studies [20, 30]. Even though preoperative planning was undertaken on digital radiography, the better exposure in the anterolateral approach still makes a difference [10], which could influence the cup position [31]. Also, the smaller 28 mm head was used throughout, whereas it has been shown that the use of a larger head decreases the dislocation rate [32].

Despite the findings, the present study has some limitations. The first is the retrospective and non-randomized design. To overcome those methodical drawbacks, the confounders between the groups have been statistically eliminated by the use of propensity score matching. Since the main indication in the vast majority of patients was osteoarthritis, indication was not a propensity score for matching. Secondly, three surgeons were involved in the study, and surgeon preference may have influenced the results. However, propensity matching using the most effective matching technique [33] ensured each patient had a similar counterpart based on age, gender, BMI and ASA score in the other groups, minimizing the surgeons’ selection bias. The follow-up for in this study was low, only looking at the in-hospital stay. Comparison on other outcomes, such as gait, was therefore not possible. It has to be noted that more than 1400 patients were included in the base population of this study. These three surgeons did the majority of the operations in the base population. Furthermore, this expresses the high degree of experience these three study surgeons have, both for the DAA and Watson Jones approaches.

## Conclusions

Direct anterior approach has comparable short-term surgical complications and reduces the transfusion and general complication rate when compared with the Watson Jones anterolateral approach.
